# Detection and genetic characterization of *Giardia duodenalis* in pigs from large-scale farms in Xinjiang, China

**DOI:** 10.1051/parasite/2019056

**Published:** 2019-08-26

**Authors:** Bo Jing, Ying Zhang, Chunyan Xu, Dongfang Li, Jinming Xing, Dayong Tao, Longxian Zhang, Meng Qi, Haiyan Wang

**Affiliations:** 1 College of Animal Science, Tarim University Alar 843300 Xinjiang PR China; 2 College of Animal Science and Veterinary Medicine, Henan Agricultural University Zhengzhou 450046 Henan PR China; 3 Experimental and Research Center, Henan University of Animal Husbandry and Economy Zhengzhou 450046 Henan PR China

**Keywords:** *Giardia duodenalis*, Prevalence, PCR, Assemblages, Zoonoses

## Abstract

To study the presence of *Giardia duodenalis* in Xinjiang, northwest China, we collected 801 fecal specimens from seven large-scale pig farms and screened them using PCR targeting the SSU rRNA gene. Twenty-one (2.6%) of the specimens from five farms were *G. duodenalis-*positive, with a significant difference in prevalence among different farms (0–8.7%) (*p* < 0.01). *Giardia duodenalis* prevalence was highest in fattening pigs (5.4%, 7/129), followed by sows (3.2%, 7/222), post-weaning piglets (1.8%, 5/281), and pre-weaning piglets (1.2%, 2/169), but there was no significant difference in prevalence among the age groups (*p* > 0.05). Sequence analysis of the SSU rRNA gene revealed that the 21 *G. duodenalis* strains belonged to three assemblages: A (*n* = 2), B (*n* = 16), and E (*n* = 3). Assemblage B was the predominant assemblage and was widely distributed in all *G. duodenalis*-positive farms and age groups. All *G. duodenalis*-positive specimens were further assayed at the β-giardin (*bg*), glutamate dehydrogenase (*gdh*), and triosephosphate isomerase (*tpi*) genes, and two *tpi*, four *gdh,* and two *bg* sequences were identified. These data indicate that pigs may be a zoonotic risk and can potentially spread *G. duodenalis* infection from animals to humans.

## Abstract (fr)

Afin d’étudier la présence de *Giardia duodenalis* dans le Xinjiang, dans le nord-ouest de la Chine, nous avons collecté 801 échantillons de selles de sept grandes exploitations porcines et les avons analysés à l’aide d’une PCR ciblant le gène SSU de l’ARNr. Vingt-et-un (2,6 %) des spécimens provenant de cinq exploitations étaient positifs pour *G. duodenalis*, avec une différence de prévalence significative entre les différentes exploitations (0 % à 8,7 %) (*p* < 0,01). La prévalence de *Giardia duodenalis* était la plus élevée chez les porcs d’engraissement (5,4 %, 7/129), suivie des truies (3,2 %, 7/222), des porcelets en post-sevrage (1,8 %, 5/281) et des porcelets en pré-sevrage (1,2 %, 2/169), mais il n’y avait pas de différence significative dans la prévalence entre les groupes d’âge (*p* > 0,05). L’analyse de la séquence du gène SSU de l’ARNr a révélé que les 21 souches de *G. duodenalis* appartenaient à trois assemblages : A (*n* = 2), B (*n* = 16) et E (*n* = 3). L’assemblage B était l’assemblage prédominant et était largement distribué dans toutes les fermes et groupes d’âge positifs pour *G. duodenalis*. Tous les échantillons positifs pour *G. duodenalis* ont ensuite été analysés pour les gènes β-giardine (*bg*), glutamate déshydrogénase (*gdh*) et triosephosphate isomérase (*tpi*), et deux séquences de *tpi*, quatre de *gdh* et deux de *bg* ont été identifiées. Ces données indiquent que les porcs peuvent présenter un risque zoonotique et potentiellement transmettre l’infection à *G. duodenalis* des animaux à l’homme.

## Introduction

*Giardia duodenalis* is a common protozoan parasite that can infect humans, livestock, companion animals, and wildlife [7]. *G. duodenalis* is considered a complex species and has been grouped into eight distinct assemblages or genotypes (A–H) based on genetic analysis. Among them, assemblages A and B have a wide host range and are responsible for the majority of known human disease cases [8], while assemblages C–H seem to be host-specific for nonhuman species (canids, domestic mammals, cats, rodents, and seals) [7]. In pigs, assemblage E is the predominant genotype in most countries, but the zoonotic assemblages A and B have also been detected in pigs, suggesting that pigs may be a reservoir for giardiasis [3, 10, 13, 20, 21].

To date, *G. duodenalis* infection has been frequently reported in a wide range of animals in China, including cattle (1.7–74.2%), sheep (0–13.1%), goats (0–27.78%), rabbits (3.9–8.3%) and other mammals (donkeys, golden takins, raccoon dogs, and horses) [11]. Although the pig industry and pig husbandry play important roles in China, few reports of *G. duodenalis* infection in pigs in China are available. In the published studies, assemblages A and E were identified in pigs, with assemblage E being the predominant assemblage [12, 20, 21]. Here, we examine the prevalence and assemblage distribution of *G. duodenalis* in pigs in the Xinjiang Uygur Autonomous Region (hereafter referred to as Xinjiang), northwest China, to assess zoonotic transmission risk and elucidate the public health significance of this protozoan parasite.

## Materials and methods

### Specimen collection

A total of 801 fresh fecal specimens were collected from 169 pre-weaning piglets (<20 days old), 281 post-weaning piglets (21–70 days old), 129 fattening pigs (71–180 days old), and 222 sows (>181 days old) from seven large-scale pigs farms in Marabishi, Alaer, Yarkant, Baicheng, Shaya, Changji, and Ruoqiang in Xinjiang between September 2017 and June 2018. Each of the sampled farms ranged from 10,000 to 80,000 pigs. These farms were visited on a single occasion and specimens were randomly collected from the animals by a veterinarian. At the time of collection, no diarrhea was apparent in the herds. Using sterile gloves, specimens were collected directly from the rectum or immediately from fresh feces deposited on the ground after animal defecation. The fresh feces were placed into clean plastic bags marked with the date, age, and farm, and immediately placed onto ice packs in an insulated container. Specimens were transported to the laboratory, stored at 4 °C, and processed no later than a week after collection.

### DNA extraction and PCR amplification

Genomic DNA was extracted from approximately 200 mg of each fecal specimen using the E.Z.N.A.R^®^ Stool DNA Kit (D4015-02, Omega Bio-Tek Inc., Norcross, GA, USA), according to the manufacturer’s instructions. Extracted DNA specimens were used as a template for polymerase chain reaction (PCR)-based analyses. Positive (dairy cattle-derived assemblage E DNA) and negative controls (distilled water) were included in each PCR assay.

*G. duodenalis* was identified using the SSU rRNA gene, as described previously [2] (Table 1). PCR reactions were conducted in 25 μL reaction mixtures consisting of 2.5 μL 1× PCR buffer (TaKaRa Shuzo Co. Ltd., Otsu, Japan), 2 μL 200 μM dNTP mixture (TaKaRa Shuzo Co. Ltd., Otsu, Japan), 0.15 μL of TaKaRa rTaq (TaKaRa Shuzo Co. Ltd., Otsu, Japan), 1.25 μL dimethyl sulfoxide (DMSO), 0.3 μM forward and reverse primer, 1 μL genomic DNA, and 17.5 μL double-distilled water. Each specimen was processed twice at the SSU rRNA gene.

**Table 1:** The primers used in the characterization of *G. duodenalis* in the present study.

Gene	Primer sequence (5′ – 3′)	Fragment length (bp)	Annealing temperature (°C)	Usage (s)	References
SSU rRNA	Gia2029: AAGTGTGGTGCAGACGGACTC	~497	55	Specific nested PCR of *G. duodenalis*	[2]
	Gia2150c: CTGCTGCCGTCCTTGGATGT				
	RH11: CATCCGGTCGATCCTGCC	~292	59		
	RH4: AGTCGAACCCTGATTCTCCGCCCAGG				
*bg*	G7: AAGCCCGACGACCTCACCCGCAGTGC	~753	65	Genotyping of *G. duodenalis*	[9]
	G759: GAGGCCGCCCTGGATCTTCGAGACGAC				
	Forward: GAACGAGATCGAGGTCCG	~511	55		
	Reverse: CTCGACGAGCTTCGTGTT				
*gdh*	Ghd1: TTCCGTRTYCAGTACAACTC	~530	50	Genotyping of *G. duodenalis*	[5]
	Gdh2: ACCTCGTTCTGRGTGGCGCA				
	Gdh3: ATGACYGAGCTYCAGAGGCACGT		50		
	Gdh4: GTGGCGCARGGCATGATGCA				
*tpi*	AL3543: AAATIATGCCTGCTCGTCG)	~605	50	Genotyping of *G. duodenalis*	[19]
	AL3546: CAAACCTTITCCGCAAACC)				
	AL3544: CCCTTCATCGGIGGTAACTT)	~530	50		
	AL3545: GTGGCCACCACICCCGTGCC)				

DNA from all SSU rRNA-positive specimens was further tested using PCRs targeting the β-giardin (*bg*), glutamate dehydrogenase (*gdh*), and triosephosphate isomerase (*tpi*) genes, as described previously [5, 9, 19] (Table 1). PCR reactions were conducted in 25 μL reaction mixtures consisting of 2.5 μL 1× PCR buffer (TaKaRa Shuzo Co. Ltd., Otsu, Japan), 2 μL 200 μM dNTP mixture (TaKaRa Shuzo Co. Ltd., Otsu, Japan), 0.15 μL of TaKaRa Ex Taq (TaKaRa Shuzo Co. Ltd., Otsu, Japan), 0.3 μM forward and reverse primer, 1 μL genomic DNA, and 18.75 μL double-distilled water. Each specimen was processed at least three times at the *bg*, *gdh* and *tpi* genes.

### Sequence analysis

PCR amplicons of the correct size were DNA sequenced by GENEWIZ (Suzhou, China). Sequence accuracy was confirmed by bidirectional sequencing. The resulting sequences were aligned against reference sequences downloaded from the National Center for Biotechnology Information GenBank database (https://www.ncbi.nlm.nih.gov/) using the ClustalX 2.1 program to determine the assemblages of *G. duodenalis* in each specimen.

All nucleotide sequences of the SSU rRNA, *bg*, *gdh,* and *tpi* genes of *G. duodenalis* isolated from pigs in this study were deposited in the GenBank database under accession numbers: MK881597 – MK881599, MK881600 – MK881601, MK881602 – MK881605, and MK881606 – MK881607, respectively.

### Statistical analysis

Differences in prevalence between ages and farms were compared with the *χ*^2^ test in SPSS for Windows (Release 13.0 standard version; SPSS Inc., Chicago, IL, USA). Differences of *p* < 0.05 were considered significant.

## Results

Of the 801 fecal specimens collected from seven farms, 21 animals (2.6%, 21/801) from five farms tested positive for *G. duodenalis* based on the SSU rRNA gene. The prevalence in pigs in this study is within the range reported in previous studies. The highest prevalence was observed in a farm from Ruoqiang (8.7%, 13/149), followed by Baicheng (5.1%, 5/99), Shaya (1.0%, 1/100), Yarkant (0.8%, 1/130), and Changji (0.8%, 1/130). *G. duodenalis* was not detected in specimens from farms in Alaer and Marabishi. The prevalence of *G. duodenalis* in pigs was significantly different among different farms (*χ*^2^ = 27.952, *df* = 5, *p* < 0.01) (Table 2). The prevalence of *G. duodenalis* in fattening pigs was 5.4% (7/129), higher than sows (3.2%, 7/222), post-weaning piglets (1.8%, 5/281), and pre-weaning piglets (1.2%, 2/169), but the differences between age groups were not significant (*χ*^2^ = 6.371, *df* = 3, *p* > 0.05) (Table 3).

**Table 2:** The prevalence and assemblages of *Giardia duodenalis* in pigs from the seven large-scale farms in Xinjiang, China.

Collection site	No. specimens	No. positive (%)	Assemblage (no.)
Marabishi	98	0	–
Alaer	95	0	–
Yarkant	130	1 (0.8)	B (1)
Baicheng	99	5 (5.1)	A (2), B (1), E (2)
Shaya	100	1 (1.0)	E (1)
Changji	130	1 (0.8)	B (1)
Ruoqiang	149	13 (8.7)	B (13)
Total	801	21 (2.6)	A (2), B (16), E (3)

**Table 3:** The prevalence and assemblages of *Giardia duodenalis* in pigs of different ages in Xinjiang, China.

Age (days)	No. specimens	No. positive (%)	Assemblage (no.)
Pre-weaning piglets (<20 days)	169	2 (1.2)	B (2)
Post-weaning piglets (21–70 days)	281	5 (1.8)	A (2), B (3)
Fattening pigs (71–180 days)	129	7 (5.4)	B (4), E (3)
Sows (>181 days)	222	7 (3.2)	B (7)

Based on the molecular analysis of the SSU rRNA gene, three assemblages, A (*n* = 2), B (*n* = 16), and E (*n* = 3), were detected among 21 *G. duodenalis*-positive specimens (Table 2). The 21 SSU rRNA-positive *G. duodenalis* specimens were further characterized based on the *tpi*, *gdh,* and *bg* genes, generating two, four, and two sequences, respectively (Table S1). Of two *tpi* sequences, one was identified as assemblage B and the other was identified as assemblage E (Table S1). The assemblage B sequence was identical to the sub-assemblage BII sequence (GenBank accession no. KX468987) from humans in Spain, while assemblage E was identified as a novel sequence and showed 99% similarity to the assemblage E sequence (KJ668134) from pigs in China. The four *gdh* sequences obtained in this study were identified as assemblages A (*n* = 1), B (*n* = 1), E1 (*n* = 1) and E2 (*n* = 1) (Table S1). Assemblage A and B sequences were identical to the sub-assemblage AII sequence (EF507661) from humans in Brazil and the assemblage B sequence from chinchillas in China, respectively. Assemblage E1 and E2 sequences were identical to the assemblage E sequence (KJ668145) from a pig in China and assemblage E sequence (MG820464) from cattle in USA, respectively. Of two *bg* sequences, one was identified as a novel sequence of assemblage B, showing 99% similarity to the assemblage B sequence (LC436571) from humans in Japan, while the other was identified as assemblage E and was identical to the assemblage E sequence (KU668892) from wild boars in China.

Based on multilocus genotyping analysis, only two fecal specimens of assemblage E and assemblage B were successfully sequenced at all three genes, forming one novel assemblage B MLG and one novel assemblage E MLG (Table S1).

## Discussion

Varying prevalence of *G. duodenalis* has been reported in pigs worldwide, ranging from 0% in pigs from Preah Vihear, Cambodia (0/74), to 66.4% (81/122) in pigs from Ontario, Canada [6, 16]. In China, the prevalence of *G. duodenalis* in domestic pigs was 1.7% (15/897) from Henan Province, 8.0% (45/560) from Shaanxi Province in domestic pigs, and 3.1% (11/357) from Sichuan Province in captive Eurasian wild boars [12, 20, 21]. In this study, 2.6% of 801 pigs were found to be infected with *G. duodenalis*. The discrepancy between previous studies was potentially due to farm hygiene management, including differences in animals stocking density, hygiene regimes, or water supply [20]. To prevent this potential issue, all pigs used in this study were from intensive breeding farms and fed using underground water. An alternative possible explanation for the transmission of *G. duodenalis* cysts is by vectors such as flies and rodents, which could be explored in future studies.

A previous study in Denmark found that the highest *G. duodenalis* prevalence was in post-weaning pigs (20–30 kg) (27.4%, 64/234), and the lowest was in the piglets (<7 weeks) (2.0%, 3/152) [14]. Similarly, a study in Australia found the highest *G. duodenalis* prevalence in post-weaning pigs (4 weeks to 6 months) (41.0%, 64/156), and the lowest in pre-piglets (11 days to 3 weeks) (18.7%, 23/123) [3]. In contrast, a study in Zambia found the highest *G. duodenalis* prevalence in sows (40.0%, 6/15), and the lowest in pre-piglets (2–5 weeks) (6.3%, 2/32) [17], and a study in Shaanxi Province, China found the highest prevalence in sows (10.5%, 6/57) and the lowest in boars (3.3%, 1/30) [20]. In this study, the highest *G. duodenalis* prevalence was in fattening pigs (70–180 days) (5.4%, 7/129), and the lowest was in pre-weaning piglets (<20 days) (1.2%, 2/169). Because of the absence of uniform age divisions and the different sample sizes across studies, it is difficult to evaluate the association between pig age and *G. duodenalis* infection; more studies should be undertaken to illustrate this association.

To date, six *G. duodenalis* assemblages (A–F) have been reported in pigs, with assemblage E being the predominant assemblage (Table 4). Among these assemblages, A, B, and E have been detected in humans. In this study, the zoonotic assemblage B was the predominant assemblage (76.0%, 16/21) and was widely distributed in all tested farms and age groups, while assemblage E was only found in three fattening pigs (Table 2). These results were consistent with a study from Ontario, Canada, where DNA sequencing detected 63 *G. duodenalis*-positive swine samples, 92.1% of which were assemblage B and 7.9% were assemblage E [6]. Previous studies in Australia [3], Denmark [10, 14], and China [20, 21], however, found that assemblage E was predominant.

**Table 4:** Global distribution of assemblages of *Giardia duodenalis* in pigs.

Location	Host	No. specimens genotyped	Genes	No. of specimens with assemblage						References
				A	B	C	D	E	F	
Denmark	Domestic pig	82	SSU rRNA and *gdh*	10			1	71		[10]
Denmark	Domestic pig	13	SSU rRNA and *gdh*	2				11		[14]
Italy	Domestic pig	1	*bg*, *tpi* and *gdh*	1						[5]
Australia	Domestic pig	55^a^	SSU rRNA	19				37	1	[3]
Canada	Domestic pig	81	SSU rRNA and *bg*		75			6		[6]
UK	Domestic pig	3	SSU rRNA			1		2		[13]
Poland	Domestic pig	5	*bg*		1			4		[18]
USA	Domestic pig	3	*bg*, *gdh* and *tpi*	2				1		[15]
China	Domestic pig	45	*bg*, *gdh* and *tpi*	9				36		[20]
China	Domestic pig	15	*tpi*	3		3		9		[21]
Nigeria	Domestic pig	53^b^	SSU rRNA and *gdh*		16			39		[1]
China	Domestic pig	21	SSU rRNA, *bg*, *gdh* and *tpi*	2	16			3		This study
Croatia	Wild boar	1	SSU rRNA, ITS and *tpi*	1						[4]
China	Wild boar	11	*bg*	2				9		[12]
Total		389	SSU rRNA, *bg*, *gdh*, ITS and *tpi*	51	108	4	1	228	1	

a Mixed infection with both assemblages A and E in two isolates.

b Mixed infection with both assemblages B and E in two isolates.

Genes *bg*, *gdh*, and *tpi* were used to determine the sub-assemblage of *G. duodenalis*. In this study, sub-assemblage AII and BII was identified at the genes *gdh* and *tpi*, respectively, which has previously been reported in humans, livestock, and companion animals worldwide [5, 7, 11]. These results reveal that pigs may play a role in human giardiasis infections. In contrast, the sub-assemblage E in this study has previously been reported in cattle, sheep and pigs.

To further clarify the genetic diversity of *G. duodenalis* in pigs, we found only one novel assemblage B MLG and one novel assemblage E MLG (Table S1), which were genetically different from previous samples from northwestern China [20]. Because there is little data on MLGs in pigs worldwide, we cannot determine the characteristics of *G. duodenalis* in pigs (such as geographic or host segregation), thus further epidemiological surveys should be undertaken to analyze the genetic differences.

## Conclusion

Although a low prevalence of *G. duodenalis* infection (2.6%, 21/801) was identified in this study, the identification of zoonotic assemblages A, B, and E, and the predominance of assemblage B, suggest that pigs pose a potential risk for the zoonotic transfer of *G. duodenali*s in the studied region.
