# Supplementary material for: Detection and genetic characterization of Giardia duodenalis in pigs from large-scale farms in Xinjiang, China
Source: Parasite. 2019 Aug 26;26:53. doi: 10.1051/parasite/2019056 (PMC6709646; doi:10.1051/parasite/2019056)
Supplement: Supplementary file 1 [file parasite-26-53-olm.pdf]

Table S1. Multilocus characterization of *Giardia duodenalis* isolates based on the *bg*, *tpi,* and *gdh* genes

Isolate	subtype			MLG type
	bg	*gdh*	*tpi*	
XJ307	B1	B1	B1	MLGB1
XJ398	-	A1	-	-
XJ435	-	E1		-
XJ558	E1	E2	E1	MLGE1
